# Micro-Abrasive Air Jet Machining Technology for Fabrication of Helical Grooves on Bovine Bone

**DOI:** 10.3390/mi16020149

**Published:** 2025-01-28

**Authors:** Jialin Li, Quanlai Li, Yafeng Deng, Weipeng Zhang, Haonan Yin

**Affiliations:** School of Computer and Artificial Intelligence, Beijing Technology and Business University, Beijing 100048, China; 2230602095@st.btbu.edu.cn (J.L.); dengyafeng@th.btbu.edu.cn (Y.D.); zhangwp@btbu.edu.cn (W.Z.); 2330602113@st.btbu.edu.cn (H.Y.)

**Keywords:** micro-abrasive air jet, bone screw, bovine bone, helical groove, material removal mechanism, groove shape

## Abstract

Biological bone screws play an important role in fixing fractures and bone defects. The machining of helical grooves on xenogenic materials is a key part of fabricating biological bone screws. The fabrication of helical grooves on bovine bone using micro-abrasive air jets was investigated in this paper. The helical groove shapes were classified and their formation mechanisms were studied. Analyses of the material removal mechanism and the effect of process parameters on the groove shapes were carried out. The results show that the helical grooves could be effectively machined using micro-abrasive air jets with a spring mask. The shapes of the helical grooves could be classified as U-, V-, and W-shaped. Cracks that propagated along the cement line may have led to the formation of a slot. Meanwhile, cracks that propagated in the interstitial lamella may have led to the formation of ridges. The slots and ridges resulted in the appearance of stripes on the groove bottom. The cracks propagated along the axial direction of the osteon at the same time as it propagated into the osteon, leading to the formation of dimples on the groove sidewall. The experimental method proposed in this study can be regarded as a suitable method to fabricate helical grooves on bones.

## 1. Introduction

Bone fractures and bone defects occur commonly in daily life. Bone screws are a type of orthopedic implant that have been widely used for fixing fractures and bone defects [1]. The prognosis of orthopedic surgery is strongly influenced by the bone screw’s mechanical and biological properties. The bone screw should have similar mechanical properties (e.g., elastic modulus, stiffness, tensile strength) to human bone, as well as good biocompatibility and high corrosion resistance [2]. Metal, bioabsorbable polymer, ceramic, and xenogenic bone can be selected to make bone screws. Metallic bone screws, which are commonly made of titanium-based alloy or stainless steel, have been widely used due to their good biocompatibility, high corrosion resistance, and ease of manufacture [3]. However, the mechanical stimuli experienced by human bone, which are near the bone screw, is reduced due to the high stiffness of metal. This effect is known as ‘stress shielding’ [4]. It increases the effects of bone resorption and reduces the effects of bone reconstruction. Hence, surgical prognosis when using metal bone screws is not good. Bioabsorbable polymer bone screws may lead to an inflammatory response and osteolytic changes [1]. Xenogenic bone screws have similar mechanical properties to human bone, as well as good biocompatibility and high corrosion resistance. They are particularly suitable for bone fracture repair and the reconstruction of bone defects [1]. As a material, bovine bone is abundant and inexpensive. As a result, bovine bone is the optimum material for making bone screws. However, bovine bone is a quasi-brittle material. When it is machined by conventional machining methods, such as milling, sawing, chiseling, and drilling, the cutting force is significant, and the bone is likely to break. Furthermore, the cutting heat is high and hence the xenogenic bone is weakened [5]. It is necessary to explore new processing methods to achieve smaller cutting force, decreased cutting heat, and higher machining efficiency.

Micro-abrasive air jet (MAAJ) machining technology, which is also known as powder blasting, was developed from traditional sand blasting [6]. During MAAJ machining, compressed air (0.2–0.7 MPa) is first mixed with a small amount of fine abrasives (average particle size of 5–200 µm) and then the mixture is expelled through a small nozzle to form a high-speed abrasive air jet beam [7]. In contrast to sand blasting, the MAAJ uses micro-abrasive, precision micro-feeding technology and hence is mainly used for micromachining. When the micro-abrasive air jet is directed toward the target, the target material will be removed under the high-speed and high-frequency impact of innumerable micro-abrasives. The cutting heat is small and is dispersed by the high-speed air stream. This method has the advantages of small cutting force, a negligible heat-affected zone, a low processing cost, and high machining efficiency [7,8]. It is suitable for processing brittle, ductile, and composite materials, including glass, steel, polymethylmethacrylate (PMMA), cement, etc.

MAAJ technology has developed rapidly in the past three decades. Nouhi et al. [9] used an MAAJ to machine helical grooves on glass and PMMA rods using a steel spring as a mask, thereby developing the surface evolution model of helical grooves. The results showed that when the particle speed was kept constant, the volume removal rate of the target material depended only on the dose of abrasives which impacted the target. It was not affected by the diameter of the rod, workpiece rotation speed, or nozzle traverse speed. By varying the spring pitch and machining parameters, helical grooves with various aspect ratios could be achieved. Luo et al. [10] employed a pair of bounding air jets to focus a central abrasive jet during the MAAJ grooving of glass. The results showed that the machined channel width could be reduced under the focused effect of the bounding air jet. Kowsari et al. [11] proposed a computational fluid dynamic-based model for predicting the erosive footprint size of planar and curved targets machined by an MAAJ. The results showed that the erosive footprint was mainly formed by the erosion of incident particles, as well as secondary erosion which was caused by rebound particles. The rebound angle of the particles varied with the surface curvature of the target, which affected the distribution of the erosive efficacy of the abrasive jet. Zhang et al. [12] developed models of individual abrasive impact velocities, impact forces, and erosion depths for the MAAJ machining of polydimethylsiloxane (PDMS). The wear mechanisms of PDMS under different impact angles were analyzed. Zhang et al. [13] fabricated V-shaped micro-channels on PDMS machined using the MAAJ direct writing method. The secondary divergence and rebound impact of the abrasive jet beam resulted in a frosted area at the edge of the micro-channel. Melentiev et al. [8] employed an MAAJ to fabricate micro-channels on a Co-Cr-Mo alloy (ASTM 1537), a type of biomedical alloy used to make hip implants. The results showed that the MAAJ was a very suitable and cost-effective means for machining micro-channels on biomedical alloys with high accuracy and high productivity. Kodama [14] employed an MAAJ to machine micro-herringbone grooves on a cylindrical workpiece, which was made of austenitic stainless steel. The result showed that the MAAJ could be used to machine micro-sloped herringbone grooves which met the design requirements. Kang [15] used an MAAJ to fabricate micro-dimples on alumina-based ceramics, and the effect of process parameters on dimple dimension was analyzed. In the work of Shafagh [6], channel networks were fabricated using an MAAJ. It was found that there were recessed dimples formed at the channel intersections, resulting in a nonuniform machining depth across the channel networks. Li et al. [16] classified the shapes of micro-holes fabricated during the MAAJ machining of glass into three categories, i.e., convex-, flat-, and concave-shaped. The particle mass distribution and the effects of particle rebound and stagnation significantly influenced the formation of micro-hole shapes. Yang et al. [17] investigated the depth of cut and material removal rate during the multi-pass radial-mode MAAJ turning of fused silica. The crossed striations on the machined surface were found.

However, to the authors’ knowledge, there is little research on the machining of helical grooves on bone materials. This paper employed fresh bovine tibia rods as the targets and used the response surface methodology (RSM) to study the microfabrication method for manufacturing helical grooves on bone using an MAAJ. The influence of process parameters on machining quality was discussed, and the material removal mechanism was analyzed. The results and analysis will be greatly helpful in understanding the bone removal mechanism during MAAJ machining and fabricating xenogenic bone screws using MAAJs.

## 2. Materials and Methods

### 2.1. Experimental Setup

**Figure 1:**
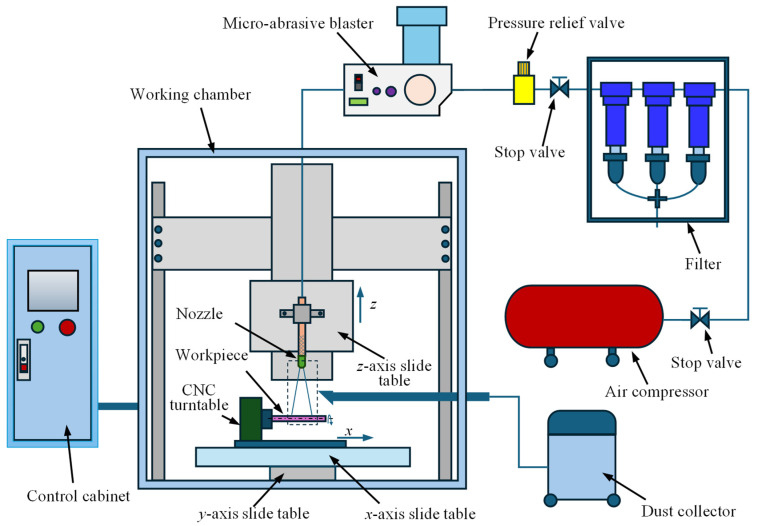
A schematic illustration of the micro-abrasive air jet turning system.

The experiments were conducted using an MAAJ turning system, as shown in Figure 1. The MAAJ turning system was equipped with an AccuFlo AF10 micro-abrasive blaster (Comco Inc., Burbank, CA, USA), an air compressor (Unical Air Compressor (Beijing) Co., Ltd., Beijing, China), a 5-axis CNC system, a filter, and a dust collector. The 5-axis CNC system consisted of 3 translational axes (*x*, *y*, and *z* axes), the rotational axis of the workpiece (*A* axis), and the axis of the workpiece inclination (*B* axis). The target was clamped by a four-jaw chuck and rotated under the control of the *A* axis. The long axis of the target was horizontal, which meant that the *B* axis was kept at 0°. The target was controlled to move in the *x* and *y* directions. The nozzle was controlled to move in the *z* direction. The concentricity of the turntable was less than 10 μm, with the end surface jumpiness being less than 15 μm. The translation positioning accuracy was ±20 μm.

### 2.2. Materials

The target material used was cortical bone, which was taken from the diaphysis section of fresh bovine tibia. Cortical bone is a brittle, transversely isotropic, and dense natural composite. It mainly consists of a circumferential lamella, interstitial lamella, and osteon. Since the structure and composition of bovine cortical bone are similar to those of human cortical bone, it has good biocompatibility and bone conductivity with the human body. Moreover, it is abundant with good processability. Hence, bovine cortical bone has a wide range of application prospects in orthopedic implants, such as bone screws and hip joints.

**Figure 2:**
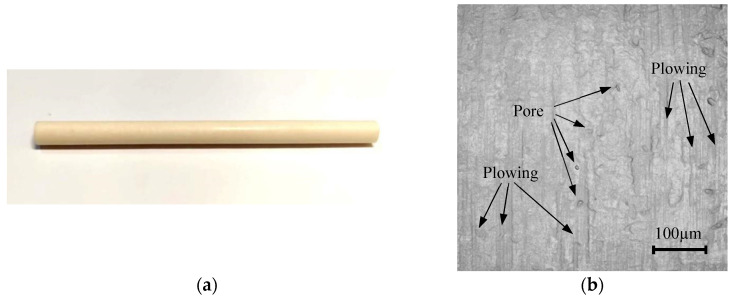
Bovine tibia cortical bone rod for MAAJ machining: (**a**) photo of prepared cortical bone rod; (**b**) surface morphology of prepared cortical bone rod.

The cortical bone of the bovine tibia was pre-turned into rods using a CNC lathe to achieve uniform initial physical dimensions and remove the circumferential lamella. In this study, bovine bone rods with a diameter of 5 mm and a length of 20 mm were selected, as shown in Figure 2a. The axis of the rod was aligned with the osteon direction. The surface morphology is shown in Figure 2b. On the target surface, there were many pores, as well as impressions formed by plowing. The arithmetical average deviation of the surface height (*R*_a_) value was 0.66 μm and the root mean square roughness (*R_q_*) value was 0.86 μm, measured along the axial direction of the rod. The mechanical properties of the target are given in Table 1. It should be noted that the strain energy release rates of the osteon, interstitial lamella, and cement line were 860, 228, and 146 N/m, respectively [18]. The fracture toughness was calculated according to [19].

(1) $$K_{\mathrm{IC}}=\sqrt{G\frac{E}{1-\gamma^{2}}}$$

where *K*_IC_ is the fracture toughness, *G* is the strain energy release rate, *E* is the elastic modulus, and *γ* is Poisson’s ratio.

**Figure 3:**
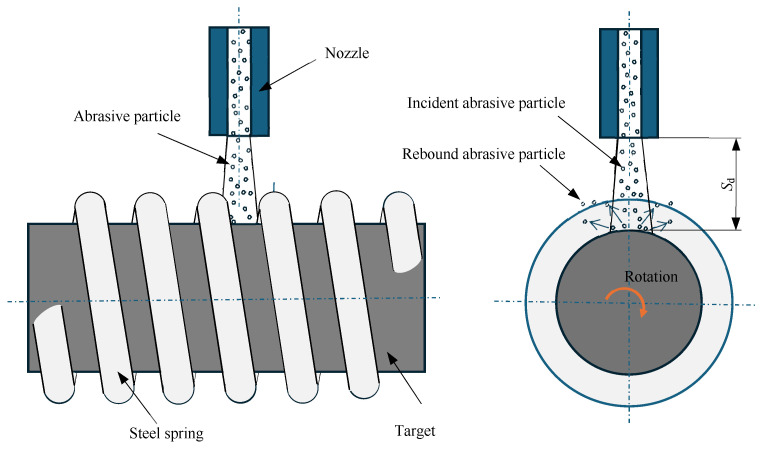
A schematic diagram of the helical groove machined by micro-abrasive air jet turning.

**Figure 4:**
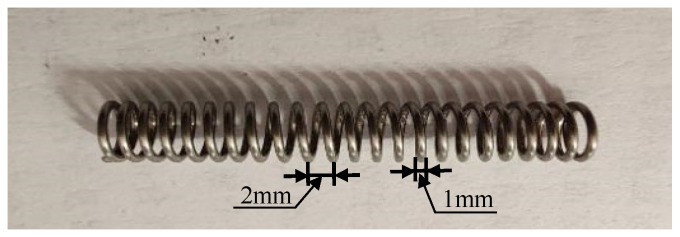
Spring mask employed for micro-abrasive air jet machining of helical grooves.

In order to machine a helical groove on the rod, a steel spring was employed as a mask and fitted onto the rod, as shown in Figure 3, which corresponds to the enlarged view of the dashed box in Figure 1. The method of machining helical grooves using a spring mask was first proposed by Nouhi et al. [9]. The thickness of the spring wire was 1 mm, as shown in Figure 4. The spring pitch, defined as the axial distance between two consecutive spring coils at the center diameter, was 2 mm. The target’s substrate beneath the spring wire was shielded by the steel spring and protected from erosion. The target’s substrate, which located in the gap between the consecutive spring coils, was exposed to the abrasive air jet and subsequently eroded.

### 2.3. Process Parameters

The process parameters considered in this study included air pressure, *P*, standoff distance, *S*_d_, nozzle traverse speed, *u*, surface velocity (target rotational surface velocity), *V*, and the number of cycles, *N*, as shown in Table 2. Air pressures were selected within common ranges of application and equipment limitations [17]. In order to avoid producing over-deep helical grooves or even cutting off the rods, the nozzle traverse speed, surface velocity, and number of cycles were appropriately adjusted. Meanwhile, it was ensured that all combinations of processing parameters could achieve significant material removal from the target. Standoff distances were selected to achieve a higher material removal rate within the determined ranges of air pressure, nozzle traverse speed, surface velocity, and the number of cycles.

Based on the central composite design (CCD) of the RSM, 52 sets of experiments were conducted. It included 32 factorial points, 10 axial points, and 10 center points. A fine nozzle with a 0.76 mm inner diameter of boron carbide was used. Angular white alumina abrasive with a nominal diameter of 50 μm was employed. The abrasive particles had a density of 3500 kg/m^3^. The calibrated abrasive flow rate was 0.54 g/min, as presented in ref. [17].

During the machining of the helical grooves, the nozzle axis intersected vertically with the target axis, resulting in a jet incidence angle of 90°. Relative to the target, the nozzle moved back and forth repeatedly along the longitudinal axis of the target. One cycle refers to the nozzle reciprocating once.

### 2.4. Measurement of Helical Grooves

To investigate the effect of process parameters on the machining quality of helical grooves, a non-contact optical 3D digital microscope (Bruker Alicona Infinite-Focus G6, Alicona Imaging GmbH, Dr.-Auner-Straße 19, 8074 Raaba, Austria) was used to measure the shape and surface roughness of the machined helical grooves on the target. The objective lens selected was the 800WD17 with a magnification of 10×. It allowed for a lateral measurement range of 1.6 mm × 1.6 mm, and the measurement point distance was 0.72 μm. The vertical resolution was 50 nm.

The surface roughness was measured following ISO 4287:1997 [20]. The arithmetical average deviation of the surface height (*R*_a_) and the root mean square roughness (*R_q_*) were selected to indicate the surface roughness of the machined helical grooves. *R*_a_ was selected due to its extensive use in the evaluation of surface roughness. It provides a general overview of the distribution of surface height. The selection of *R*_a_ as a parameter for surface roughness evaluation was also beneficial for the comparison of the results in this study with existing research findings. *R_q_* is more sensitive to larger deviations of surface height than *R*_a_. As for micro-abrasive air jet-machined surfaces, it is common to find that different machined surfaces have nearly identical *R*_a_ values while exhibiting clearly different *R_q_* values. In order to effectively distinguish the surfaces with nearly identical *R*_a_ values while exhibiting clearly different *R_q_* values, both *R*_a_ and *R_q_* were selected in this study.

During micro-abrasive air jet machining, there are various fine structures formed on the machined surface, such as dimples, small craters, scratches, and so on. In order to characterize these fine structures, the cut-off length was selected as 0.25 mm with an evaluated length of 2 mm.

## 3. General Assessment of Machining Quality

### 3.1. Morphology of Helical Grooves

**Figure 5:**
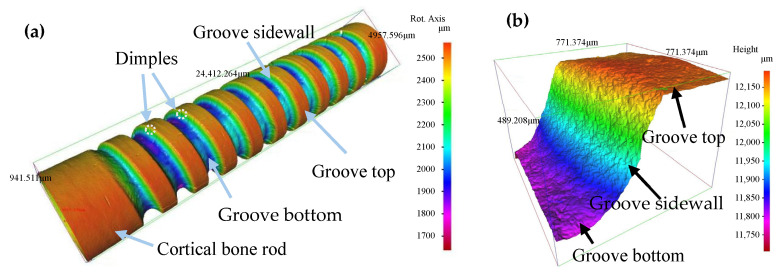

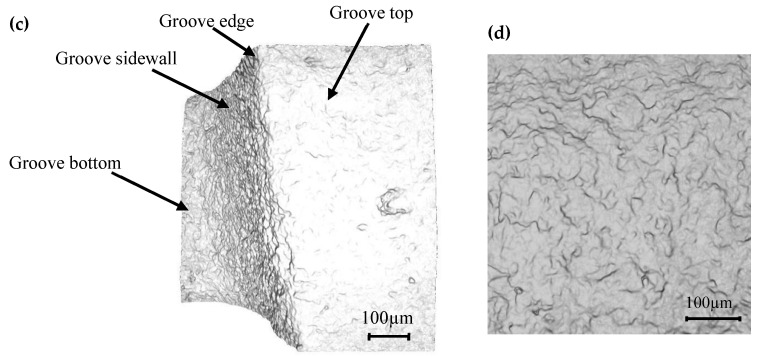
Helical groove machined by micro-abrasive air jet (*P* = 0.6 MPa; *S*_d_ = 1.5 mm; *u* = 0.54 mm/s; *V* = 7.85 mm/s; *N* = 10): (**a**) 3D microscopic photograph of helical groove; (**b**) 3D morphology of groove sidewall; (**c**) microscopic photograph of groove edge; (**d**) microscopic photograph of groove bottom.

In order to evaluate the capabilities of MAAJ machining for fabricating helical grooves on bone material, general microscopic observations of the helical grooves were carried out first. Typical microscopic photographs of a helical groove machined in the experiment are shown in Figure 5. It can be seen from Figure 5a that there are no macroscopic cracks on the groove surface. There are dimples on the helical groove sidewall. Figure 5b shows that the surface of the groove sidewall is relatively rough. Figure 5c provides a top view of Figure 5b. It can be seen from Figure 5c that the groove edge is not sharp, which is similar to the machining edges of brittle materials machined by MAAJs [21]. Ghobeity et al. [22] reported that the distribution of particle kinetic energy is nonuniform across the cross-section of the abrasive air jet. Based on statistical analysis, the particle kinetic energy at the rim of the jet is lower than that at the center. At the rim of the jet, some particles have higher kinetic energy and can remove the target material immediately, while others have lower kinetic energy and only weaken the target material. Moreover, Ghobeity et al. [22] also reported that the mass distribution of the abrasive particles is nonuniform across the cross-section of the abrasive air jet. The particles at the rim of the jet are fewer than those in the region close to the center of the jet. The dispersed particles at the rim may result in a discontinuous impact impression. Hence, the groove edge, which consists of a damaged area, is irregular and unclear. It can be seen from Figure 5d that the groove bottom is rough; however, the machining texture is not found. The impression of plowing is not found on either the groove sidewall or the groove bottom. This indicates that the ductile removal mechanism is not the dominant method of material removal. Since the cortical bone is brittle material, the target may be mainly removed through brittle fracture, and hence texture and plowing cannot be found.

The surface roughness of 52 experimental sets was evaluated. As for the groove sidewall, the arithmetical average deviation of the surface height (*R*_a_) and the root mean square roughness (*R_q_*) were measured along the circumferential direction of the helical groove. The results show that the *R*_a_ values ranged from 0.76 μm to 5.12 μm, and the *R_q_* values ranged from 1.07 μm to 7.32 μm. As for the groove bottom, the *R*_a_ values ranged from 1.82 μm to 4.05 μm, and the *R_q_* values ranges from 2.23 μm to 5.6 μm, along the axial direction of the target.

### 3.2. Typical Shapes of Helical Grooves

**Figure 6:**
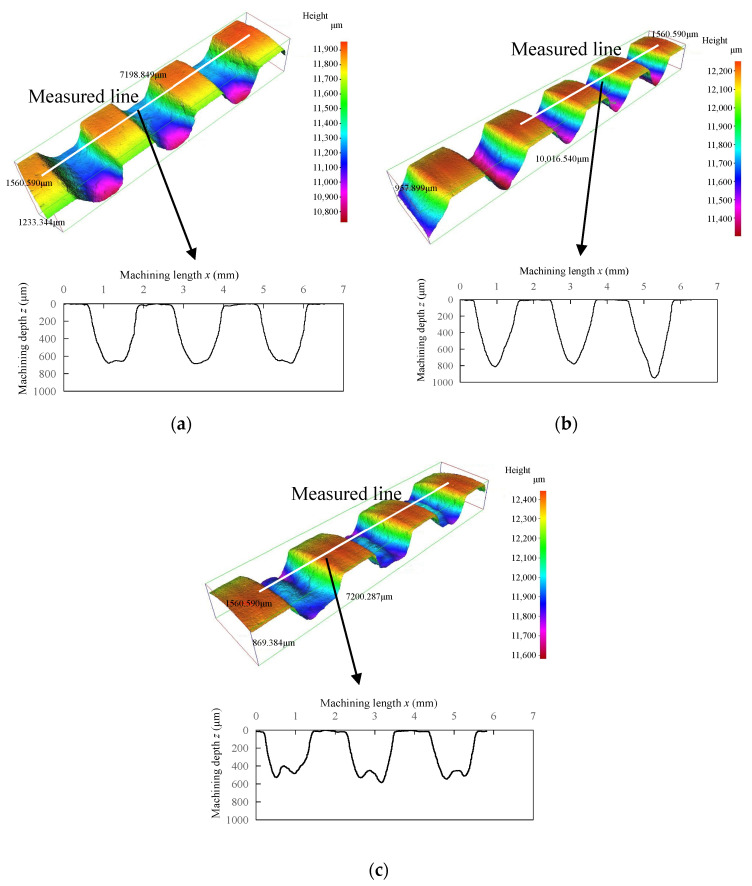
Typical shapes of helical grooves: (**a**) U-shaped helical groove (*P* = 0.7 MPa; *S*_d_ = 1 mm; *u* = 0.27 mm/s; *V* = 10.46 mm/s; *N* = 8); (**b**) V-shaped helical groove (*P* = 0.6 MPa; *S*_d_ = 1.5 mm; *u* = 0.54 mm/s; *V* = 7.85 mm/s; *N* = 8); (**c**) W-shaped helical groove (*P* = 0.5 MPa; *S*_d_ = 1 mm; *u* = 0.27 mm/s; *V* = 5.23 mm/s; *N* = 12).

The typical shapes of the helical grooves are illustrated in Figure 6. It can be seen from the figure that the shape of helical grooves machined by the MAAJ could be classified into three categories. Firstly, there were U-shaped helical grooves with a bowl-shaped bottom. Secondly, there were V-shaped helical grooves with an almost pointed bottom. Lastly, there were W-shaped helical grooves with a concave–convex bottom. The percentages of the numbers of the various types of grooves with respect to the total number of grooves were 71%, 14%, and 15%, respectively.

**Figure 7:**
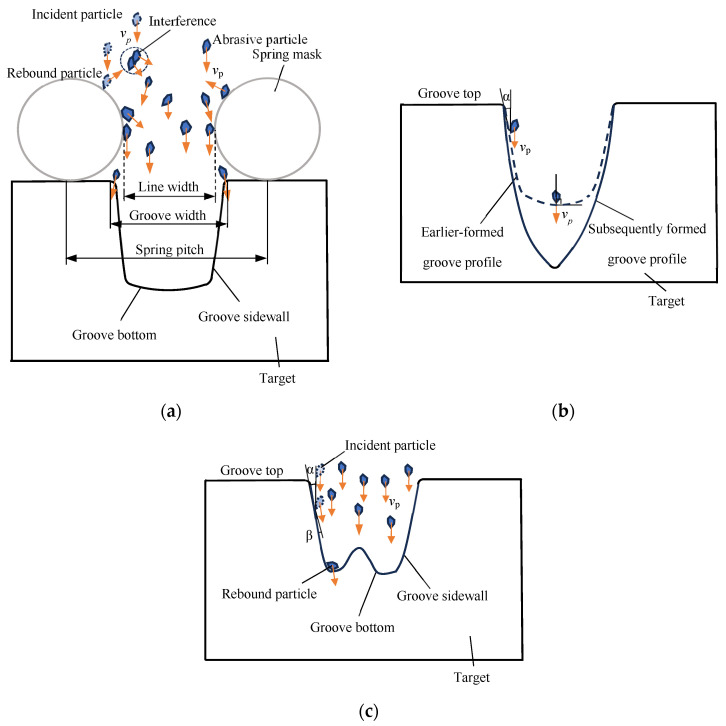
Schematic diagram of typical helical groove shapes: (**a**) schematic diagram of U-shaped helical groove; (**b**) schematic diagram of V-shaped helical groove; (**c**) schematic diagram of W-shaped helical groove. (Orange arrow indicates direction of abrasive particle velocity).

The shape of the particles was irregular, and their size was not uniform. As a result, when the abrasive jet passed through the spring mask, the particles near the spring wire were likely to be blocked by it. The amount of particles that could impact the marginal region of the groove was reduced. Furthermore, the incident particles near the spring wire were rebounded by the spring wire and interference with subsequent incident particles, as shown in Figure 7a. It may have resulted that the amount of particles that could impact the marginal region of the groove was further reduced. In the marginal region of the groove, fewer particles impacting it may have led to less material removal and a shallower machining depth. Conversely, the particles that impacted the center area of the groove were far away from the spring wire. The blocked effect of the spring wire was negligible. Hence, in the center area of the groove, more particles impacting it may have resulted in more material removal and a deeper machining depth. As a result, U-shaped helical grooves with sloping sidewalls and bowl-shaped groove bottoms were generated.

Once the sloping sidewall (indicated by the dashed line in Figure 7b) was formed, the impact angle of the particle (the angle between the particle’s velocity direction and the tangent direction of the target surface at the impact site), α, on the sloping sidewall was smaller than that on the bowl-shaped groove bottom. Since cortical bone is a brittle material, a particle with a larger impact angle resulted in greater material removal. Therefore, the sloping sidewall led to less material removal. As processing progressed, the sloping sidewall gradually became steeper and its area increased. In contrast, the area of the bowl-shaped bottom gradually decreased. Once the two sidewalls nearly intersected at the groove bottom, a V-shaped helical groove was formed, as shown in Figure 7b.

The formation of W-shaped helical grooves may be attributed to rebound particles. When an incident particle impacted the groove sidewall, it rebounded. The rebound angle of the particle, β, was smaller than the impact angle of the incident particle, α [23]. Hence, it rebounded toward the marginal region of the groove bottom. The region was also near the groove sidewall due to the small rebound angle. If the rebound particle retained sufficient enough kinetic energy, it may have caused material removal from the target through the so-called “particle secondary strike” effect [24]. Therefore, more material removal in the marginal region of the groove bottom may have led to the generation of a W-shaped helical groove, as shown in Figure 7c.

### 3.3. Dispersion of Helical Groove Width

Along the measured line, three continuous cross-sections of each helical groove were recorded, as shown in Figure 6. Hence, the maximum groove width (*G*_max_), minimum groove width (*G*_min_), and arithmetic mean of the groove width (*G*_ave_) of each helical groove could be determined.

**Figure 8:**
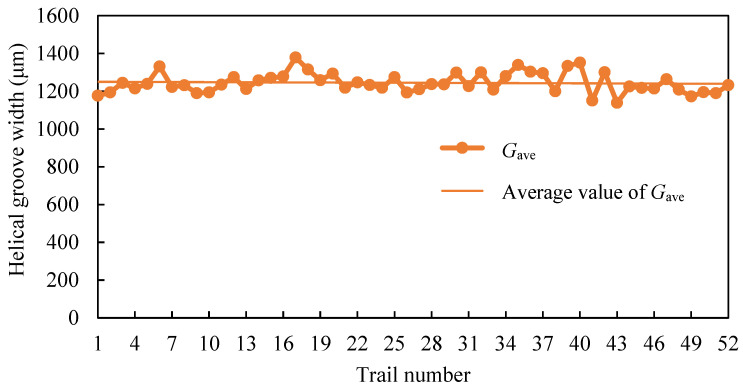
Average helical groove width of target.

*G*_ave_ for the 52 sets of experiments is shown in Figure 8. It can be seen that the groove width varied only within a narrow range. This indicates that the process parameters had an insignificant effect on the groove width. The groove width may have been primarily influenced by the spring pitch. The groove width was larger than the spring pitch. This is because some particles struck the marginal region of the groove at an oblique angle, as shown in Figure 7a. As a result, the groove width was larger than the spring pitch.

To investigate the controllability of the MAAJ process and the quality of machining, the degree of width dispersion for each helical groove was calculated as

*η* = (*G*_max_ − *G*_min_)/*G*_ave_ (2)

where *η* is degree of width dispersion.

In this study, *η* was in the range of 1–9.4%, and the average value of *η* was 5.41% across all targets. This demonstrates that MAAJ machining could effectively control the width of the helical groove for bovine tibia cortical bone by simply employing a steel spring mask.

## 4. Material Removal Mechanism Analysis of Cortical Bone

**Figure 9:**
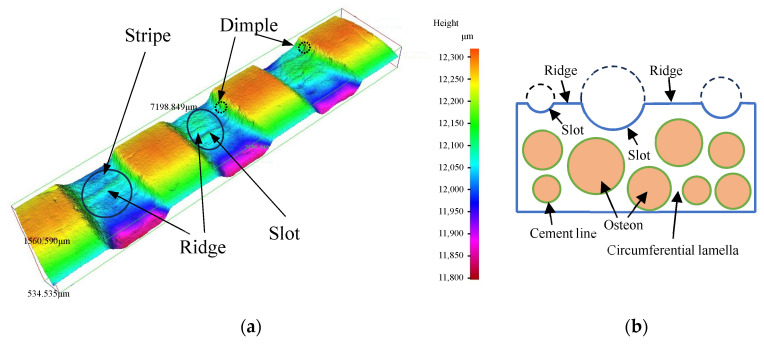
Material removal mechanism analysis of cortical bone. (**a**) The morphology of the stripe and dimple on the helical groove (*P* = 0.7 MPa; *S*_d_ = 1 mm; *u* = 0.81 mm/s; *V* = 10.46 mm/s; *N* = 12). (**b**) The formation mechanism of the ridge and slot.

It can be observed from Figure 9a that there are stripes with ridges and slots on the groove bottom. The orientation of these stripes is parallel to the axis of the target. Additionally, dimples are visible on the groove sidewall. Some of the dimples connect to the slots. To the authors’ knowledge, these distinct features on the groove surface of bone material machined by an MAAJ are reported here for the first time. To identify the reasons for the presence of distinct features, an analysis of the material removal mechanism was conducted.

In this study, the axis direction of the target aligned with that of the osteon. An osteon is the primary structure unit of cortical bone, characterized as a cylindrical unit with length of 3–5 mm and diameter of 50–300 µm [25]. Each osteon is surrounded by the cement line, which is a thin layer with a thickness of 1–5 µm [25]. The interstitial lamella occupies the remaining space within cortical bone. Among the osteon, interstitial lamella, and cement line, the cement line represents a weak zone with the lowest toughness, as discussed in Section 2.2. Hence, when a crack nucleated near the osteon, it tended to propagate in the cement line. This indicated that the crack was likely to propagate circumferentially and axially along the cylindrical surface of the osteon, resulting in larger pieces of osteon fragment being removed. As a result, slots were formed, as shown in Figure 9b. Conversely, when a crack nucleated in an area far away from the osteon, it tended to propagate within the interstitial lamella. Given that the toughness of the interstitial lamella is greater than that of the cement line, the length of crack propagation in the interstitial lamella was shorter than that in the cement line. This resulted in the removal of smaller interstitial lamella fragments and the formation of ridges. This phenomenon is analogous to the study of the orthogonal cutting of bone, where the direction is transverse to the osteon orientation [25].

Although the osteon exhibited the highest toughness and had the shortest crack propagation length, microcracks may have been formed and propagated into the osteon. The accumulation of these microcracks may have also led to transverse fractures in the osteon. Since the toughness of the cement line was lower than that of the osteon, cracks also propagated within the cement line and along the axial direction of the osteon at the same time as they propagated into the osteon. As a result, dimples were generated on the groove sidewall.

The stripe morphology varied as the machining process progressed. The osteon may have been present in a different position, leading to distinct stripe morphologies at different machining depths. Regarding the circumferential lamella in the ridge, the impact angle of the particle was smaller when it struck the slot. Conversely, the impact angle of the particle was higher when it struck the ridge. Since the circumferential lamella was brittle, a higher particle impact angle resulted in a greater target material removal rate. This meant that the ridge was removed more than the slot, causing the stripe to become less pronounced. When another osteon, situated in the target substrate, was impacted by the particle, cracks may have nucleated and propagated. The crack length along the circumference of the cement line increased with an increase in the number of particles impacting the osteon. The osteon may have peeled up, leading to the formation of a new slot. Hence, a new stripe was generated.

**Figure 10:**
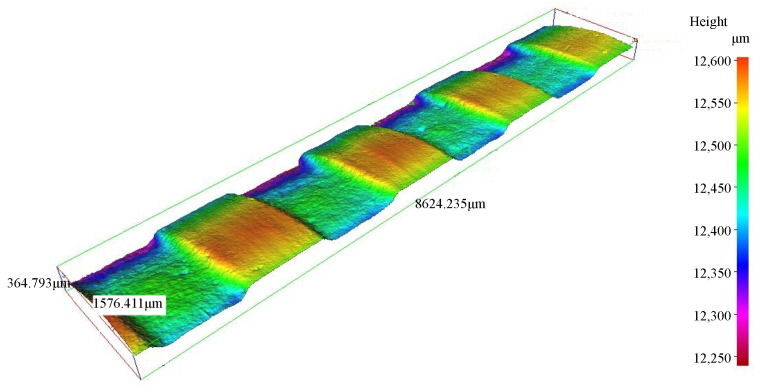
Helical groove without significant stripes and dimples (*P* = 0.5 MPa; *S*_d_ = 2 mm; *u* = 0.27 mm/s; *V* = 10.46 mm/s; *N* = 8).

It should be noted that the stripes and dimples were insignificant on some of the grooves, as illustrated in Figure 10. Regarding the stripes, similarly to the analysis of variations in stripe morphology, the disappearance of stripes may be attributed to differences in material removal rates for ridges and slots. Concerning the dimples, the particle impact angle on the groove sidewall was small. A smaller particle impact angle resulted in less material removal. Consequently, the uneven morphology on the groove sidewall may have been removed without significantly altering the shape of the groove sidewall. Hence, the dimples were insignificant on the groove sidewall.

## 5. Effect of Process Parameters on Helical Groove Shape

In order to investigate the effect of process parameters on the shape of helical grooves and to recommend an optimal selection of process parameters, main effect analyses were conducted.

### 5.1. Effect of Process Parameters on Groove Depth

**Figure 11:**
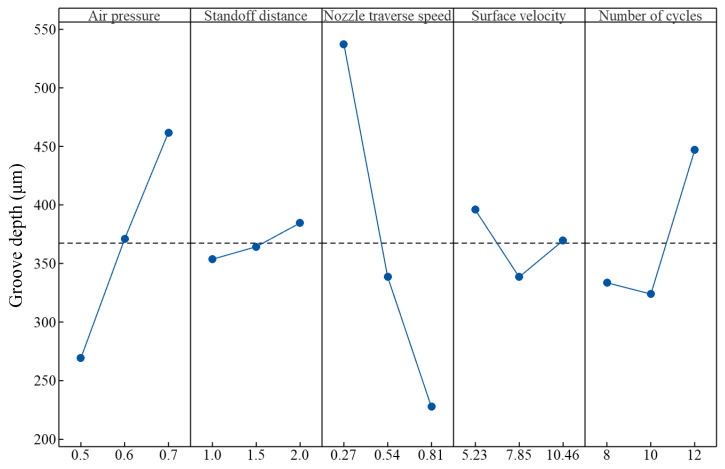
Main effect of process parameters on groove depth.

The main effects of process parameters on groove depth are shown in Figure 11.

The groove depth increased with an increase in air pressure. Stevenson et al. [26] found that the square of particle velocity is directly proportional to air pressure, that is,

(3) $$v_{p}^{2}\propto \frac{P}{d_{n}^{0.57}}$$

where *v_p_* is the particle velocity and *d_n_* is the inner diameter of the nozzle.

The kinetic energy of a particle can be expressed by

(4) $$U_{p}=\frac{1}{2}m_{p}v_{p}^{2}$$

where *U_p_* is the particle kinetic energy and *m_p_* is the mass of the particle.

Therefore, the kinetic energy of a particle is proportional to air pressure. Higher air pressure resulted in greater particle kinetic energy, leading to more intense collisions between a particle and cortical bone. This could result in the nucleation of more cracks and larger cracks. Consequently, more target material was removed and a larger groove depth was obtained.

The groove depth increased with an increase in standoff distance. The particle velocity increased first and then decreased once it was ejected from the nozzle [27]. Li [27] reported that when the particle flight distance along the nozzle axial direction is less than the critical distance, which is 6.2*d_n_*, the particle velocity keeps increasing. The critical distance is 4.7 mm for a *d_n_* of 0.76 mm. In this study, the standoff distance ranged from 1.0 to 2.0 mm, which was below the critical distance. Hence, the particle velocity increased with an increase in standoff distance. This resulted in the increase in particle kinetic energy and groove depth. In addition, the dispersion of the abrasive jet beam may have also increased with an increase in standoff distance [27]. The interference among particles in the abrasive jet may have decreased, resulting in an increase in the effective kinetic energy of the abrasive jet acting on the cortical bone, and hence the groove depth increased. Furthermore, incident particles, which impacted the groove sidewalls, rebounded and may have impacted the groove bottom, creating a secondary strike effect, as discussed in Section 3.2. The particle secondary strike effect contributed to the removal of target material and the increase in groove depth. It should be noted that the effect of standoff distance on groove depth was insignificant.

The groove depth decreased with an increase in nozzle traverse speed. The machining time of the micro-abrasive air jet decreased with an increase in nozzle traverse speed. As a result, the number of particles impacting the target material decreased. This caused the material removal amount to decrease, and hence the groove depth decreased.

The groove depth decreased first and then increased with an increase in surface velocity. On one hand, the number of particles impacting a specific area of the target surface decreased with an increase in surface velocity, meaning that the kinetic energy of the abrasive air jet impacting that area of the target surface decreased. Correspondingly, the material removed decreased, leading to a decrease in groove depth. On the other hand, since the number of particles impacting a specific area of the target surface decreased, the interferences of particles may have also decreased. Therefore, the effective kinetic energy of the abrasive air jet may have increased, and hence the groove depth increased. As for the surface velocity increase from 5.23 to 7.85 mm/s, the effect of the former may have been greater than that of the latter, and hence the groove depth decreased. Regarding the surface velocity increase from 7.85 to 10.46 mm/s, the effect of latter may have been greater than that of the former, and hence the groove depth increased. Under the combined effect of the above two aspects, the groove depth decreased first and then increased with an increase in surface velocity.

The groove depth remained relatively stable first and then increased with an increase in the number of cycles. As for the number of cycles of 8, 10, and 12, the groove depths were 333.4, 323.8, and 447.2 μm, respectively. As the number of cycles varied from 8 to 10, the variation in groove depth was only 2.8%. This indicates that the effect of the number of cycles on groove depth was insignificant in this case. The difference in groove depth may be attributed to the measurement error, which arose from the selection of positions at the groove top and bottom. As the number of cycles varied from 10 to 12, the variation in groove depth was 36.1%, indicating that the effect of the number of cycles on groove depth was significant in this case. The number of particles impacting the target increased as the number of cycles varied from 8 to 10. This contributed to an increase in groove depth. However, the impact angle of the abrasive particles reduced due to the inclined sidewall of the groove. For brittle materials, the machining ability of particles decreases with a decrease in the particle impact angle [28]. Hence, the material removal rate decreased when the number of cycles varied from 8 to 10. Considering the combined effects of the above two aspects, the variation in the groove depth was negligible. It should be noted that, although the variation in material removal was insignificant, the damage received by the target substrate caused by particle impact may have significantly increased and accumulated. When the number of cycles varied from 10 to 12, the damaged substrate material could be removed easily, resulting in a significant increase in groove depth.

The analysis above indicates that a greater groove depth could be achieved by increasing the air pressure and standoff distance, as well as decreasing the nozzle traverse speed, within the operating range employed in this study. Moreover, a lower surface velocity and a larger number of cycles also contributed to obtaining a larger groove depth.

### 5.2. Effect Process Parameters on Groove Width

**Figure 12:**
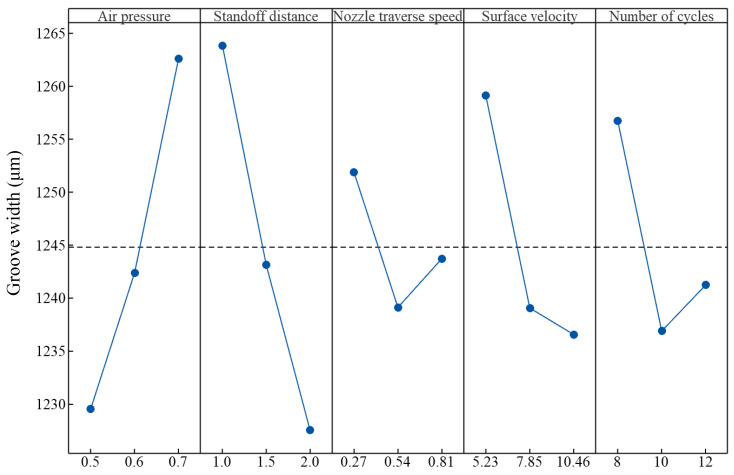
Main effects of process parameters on groove width.

The main effects of process parameters on groove width are shown in Figure 12. For air pressure, standoff distance, nozzle traverse speed, surface velocity, and the number of cycles, the maximum percentage differences in groove width were 2.6%, 2.9%, 1%, 1.8%, and 1.6%, respectively. This indicates that the process parameters had insignificant effects on the groove width, which is consistent with the discussion presented in Section 3.3.

The groove width increased with an increase in air pressure. Since the particle kinetic energy increased with an increase in air pressure, the kinetic energy of particles impacting near the margin region of the groove also increased. Hence, the groove width increased.

The groove width decreased with an increase in standoff distance. As discussed in Section 5.1, the particle kinetic energy and the dispersion of the abrasive jet increased with an increase in standoff distance. On one hand, an increase in particle kinetic energy may have resulted in an increase in the groove width. On the other hand, an increase in the dispersion of the abrasive jet resulted in a larger jet footprint. The number of particles that could pass through the spring mask decreased. Correspondingly, the number of particles impacting the marginal region of the groove decreased. The groove width may have decreased. The effect of the latter may have been greater than that of the former. Hence, the groove width decreased with an increase in standoff distance.

For nozzle traverse speed, surface velocity, and the number of cycles, the maximum percentage differences in the groove width was less than 2%. The effects of nozzle traverse speed, surface velocity, and the number of cycles may be assumed to be negligible. The slight variation in the groove width may be primarily attributed to the measurement error arising from selection of the groove edge position.

The analysis above shows that the effect of the five aforementioned process parameters on groove width was small when compared to the spring pitch. To control groove width, air pressure and standoff distance are major concerns, while a higher nozzle traverse speed, a higher surface velocity, and a lower number of cycles may be used from an economic point of view.

## 6. Conclusions

In this paper, experiments on helical groove fabrication on bovine bone using micro-abrasive air jets were carried out. The groove shapes were classified and their formation mechanisms were studied. The analysis of the material removal mechanism and the effect of process parameters on groove shape were carried out. The conclusions are as follows:

Helical grooves could be effectively machined on bovine bone using a micro-abrasive air jet with a spring mask. The groove shapes were clear and there were no macroscopic cracks or plowing on the groove surfaces. The results showed that, regarding the groove sidewall, the *R*_a_ value was 0.76 μm–5.12 μm, and the *R*_q_ value was 1.07 μm–7.32 μm. Regarding the groove bottom, the *R*_a_ value was 1.82 μm–4.05 μm, and the *R*_q_ value was 2.23 μm–5.6 μm, along the axial direction of the target.

The shape of the helical grooves could be classified into three categories, i.e., U-shaped, V-shaped, and W-shaped helical grooves. The percentages of the numbers of the various types of grooves with respect to the total number of grooves were 71%, 14%, and 15%, respectively. The formation of U-shaped helical grooves may be attributed to the blocked effect of the spring wire, which resulted in fewer particles impacting the marginal region of the groove compared to the central region. The formation of V-shaped helical grooves was primarily due to a decrease in the particle impact angle at the groove sidewall. The formation of W-shaped helical grooves may be attributed to the secondary strike effect caused by rebound particles in the marginal region of the groove bottom.

There were stripes consisting of slots and ridges on the groove bottom, as well as dimples on the groove sidewall. Based on the composition of cortical bone, the formation mechanism of the stripes on the groove bottom and the dimples on the groove sidewall were analyzed. Cracks caused by the impact of particles were likely to propagate along the cement line. This led to the removal of larger osteon fragments, and a slot was formed. The length of crack propagation in the interstitial lamella was shorter than that in the cement line, which resulted in the formation of ridges. The crack propagated along the axial direction of the osteon at the same time as it propagated into the osteon, resulting in the formation of dimples on the groove sidewall. The stripe morphology varied as the machining process progressed, and grooves without significant stripes or dimples could be formed.

The groove depth increased with an increase in air pressure and standoff distance. The groove depth decreased with an increase in nozzle traverse speed. The groove depth decreased first and then increased with an increase in surface velocity and the number of cycles. The groove width was primarily affected by the spring pitch. The groove width increased with an increase in air pressure and the groove width decreased with an increase in standoff distance. The effect of nozzle traverse speed, surface velocity, and the number of cycles on the groove width was insignificant.

The results showed that the proposed experimental method can be regarded as a suitable method to fabricate helical grooves on bones. However, there are various types of bone screws that are designed for different application requirements. Correspondingly, the MAAJ machining process may also be different when fabricating different types of bone screws. The processing efficiency and cost are closely related to the selected processing technology. Future work should be conducted to optimize the processing technology for various types of bone screws, as well as to evaluate their processing efficiency and cost.

## Figures and Tables

**Table 1:** Mechanical properties of the target material [18].

	Osteon	Interstitial Lamella	Cement Line
Elastic modulus (GPa)	20.7	22.8	6.85
Fracture toughness (MPa·m^1/2^)	4.3	2.31	1.14
Poisson’s ratio	0.18	0.16	0.48
Density (kg/m^3^)	2000	2000	2000

**Table 2:** Process parameters of the experiment.

Process Variables	Level 1	Level 2	Level 3
Air pressure, *P* (MPa)	0.5	0.6	0.7
Standoff distance, *S*_d_ (mm)	1	1.5	2
Nozzle traverse speed, *u* (mm/s)	0.27	0.54	0.81
Surface velocity, *V* (mm/s)	5.23	7.85	10.46
Number of cycles, *N* (times)	8	10	12

## Data Availability

The data presented in this study are available on request from the corresponding author.

## References

[1] Heo S.Y., Kim N.S. (2014). Comparison of the bone response to xenogenic bone screws and metallic screws in canine femur. Vet. Med..

[2] Rouhi G., Tahani M., Haghighi B., Herzog W. (2015). Prediction of stress shielding around orthopedic screws: Time-dependent bone remodeling analysis using finite element approach. J. Med. Biol. Eng..

[3] Luo J., Tamaddon M., Yan C., Ma S., Wang X., Zhou F., Liu C. (2020). Improving the fretting biocorrosion of Ti6Al4V alloy bone screw by decorating structure optimised TiO_2_ nanotubes layer. J. Mater. Sci. Technol..

[4] Haase K., Rouhi G. (2013). Prediction of stress shielding around an orthopedic screw: Using stress and strain energy density as mechanical stimuli. Comput. Biol. Med..

[5] Shu L., Sugita N. (2020). Analysis of fracture, force, and temperature in orthogonal elliptical vibration-assisted bone cutting. J. Mech. Behav. Biomed. Mater..

[6] Shafagh S., Papini M. (2020). The effects of blast lag in abrasive jet machined micro-channel intersections. Precis. Eng..

[7] Wang J., Moridi A., Mathew P. (2011). Micro-grooving on quartz crystals by an abrasive air jet. Proc. Inst. Mech. Eng. Part C J. Mech. Eng. Sci..

[8] Melentiev R., Fang F. (2020). Fabrication of micro-channels on Co-Cr-Mo joints by micro-abrasive jet direct writing. J. Manuf. Process..

[9] Nouhi A., Spelt J.K., Papini M. (2018). Abrasive jet turning of glass and PMMA rods and the micro-machining of helical channels. Precis. Eng. J. Int. Soc. Precis. Eng. Nanotechnol..

[10] Luo X., Palumbo J., Papini M., Spelt K. (2019). Aerodynamic focusing of an abrasive air jet and its effect on machining resolution. Int. J. Mach. Tools Manuf..

[11] Kowsari K., Nouhi A., Hadavi V., Spelt J.K., Papini M. (2017). Prediction of the erosive footprint in the abrasive jet micro-machining of flat and curved glass. Tribol. Int..

[12] Zhang G., Sun Y., Gao H., Zuo D., Liu X. (2021). A theoretical and experimental investigation of particle embedding and erosion behaviour of PDMS in micro-abrasive air-jet machining. Wear.

[13] Zhang G., Sun Y., Gao H., Liu X., Zuo D. (2022). Characteristics of cryogenic abrasive air-jet direct-write machining: A comparison with abrasive air-jet direct-write machining at oblique angles. J. Mater. Process. Technol..

[14] Kodama H., Nakamae S., Harada M., Wada D., Ohashi K. (2021). Abrasive jet machining for the microprofile control patterning of herringbone grooves. Precis. Eng..

[15] Kang C., Liang F., Shen G., Wu D., Fang F. (2021). Study of micro-dimples fabricated on alumina-based ceramics using micro-abrasive jet machining. J. Mater. Process. Technol..

[16] Li H., Wang J., Kwok N., Thai N., Yeoh G.H. (2018). A study of the micro-hole geometry evolution on glass by abrasive air-jet micromachining. J. Manuf. Processes.

[17] Yang R., Li Q., Zhang W., Deng Y., Li J. (2023). An Investigation into the multi-pass radial-mode micro abrasive air jet turning of fused-silica rods. Machines.

[18] Sugita N., Shu L., Shimada T., Oshima M., Kizaki T., Mitsuishi M. (2017). Novel surgical machining via an impact cutting method based on fracture analysis with a discontinuum bone model. CIRP Ann..

[19] Ewalds H.L., Wanhill R.J.H. (1989). Fracture Mechanics.

[20] (1997). Geometrical Product Specifications (GPS)—Surface Texture: Profile Method—Terms, Definitions and Surface Texture Parameters.

[21] Li Q.L., Huang C.Z., Wang J., Zhu H.T., Liu Z.W. (2010). An experimental study on the kerf characteristics of silicon cut with micro abrasive air jet. Adv. Mater. Res..

[22] Ghobeity A., Krajac T., Burzynski I., Papini M., Spelt J.K. (2008). Surface evolution models in abrasive jet micromachining. Wear.

[23] Belloy E., Sayah A., Gijs M.A.M. (2001). Oblique powder blasting for three-dimensional micromachining of brittle materials. Sens. Actuat. A-Phys..

[24] Slikkerveer P.J., Veld F.H.i.t. (1999). Model for patterned erosion. Wear.

[25] Liao Z., Axinte D.A. (2016). On chip formation mechanism in orthogonal cutting of bone. Int. J. Mach. Tools Manuf..

[26] Stevenson A.N.J., Hutchings I.M. (1995). Scaling laws for particle velocity in the gas-blast erosion test. Wear.

[27] Li H.Z., Wang J., Fan J.M. (2009). Analysis and modelling of particle velocities in micro-abrasive air jet. Int. J. Mach. Tools Manuf..

[28] Li Q., Deng Y., Li J., Shi W. (2020). Roughness characterization and formation mechanism of abrasive air jet micromachining surface studied by power spectral density. J. Manuf. Process..

